# Comparative analysis of the pedicle screw accuracy, screw revision and loosening rate and radiation exposure of robotic-guided (RG), intraoperative computed tomography (iCT)-navigation guided, and fluoroscopy guided placement technique

**DOI:** 10.1016/j.bas.2025.105899

**Published:** 2025-12-04

**Authors:** Mirza Pojskić, Miriam Bopp, Omar Alwakaa, Christopher Nimsky, Benjamin Saß

**Affiliations:** a Department of Neurosurgery, University of Marburg, Marburg, Germany; b Center for Mind, Brain and Behavior (CMBB) Marburg, Germany

**Keywords:** Intraoperative computed tomography, Fluoroscopy-guided pedicle screw placement, Navigation-guided pedicle screw placement, Robotic-guided pedicle screw placement, Pedicle screw placement accuracy, Radiation exposure in spine surgery

## Abstract

**Objective:**

This retrospective single-center study aimed to compare the accuracy and revision rates of pedicle screw (PS) placement using robot-guided (RG), intraoperative CT-navigated (iCT-nav), and fluoroscopy-guided (FG) techniques. Additionally, screw loosening and overall revision rates were assessed across all three methods.

**Methods:**

Data from 237 consecutive patients who underwent PS placement using iCT-nav, FG, or RG were analyzed. Each PS was evaluated in intraoperative or postoperative CT and classified using the Gertzbein-Robbins Scale (GRS). Follow-up CT to assess fusion and screw loosening was performed at a median of 8 months (IQR = 5–17).

**Results:**

A total of 1352 PS were placed: 444 with RG, 667 with FG, and 241 with iCT-nav. RG showed the highest rate of GRS A screws (91.7 %) compared to iCT-nav (86.2 %) and FG (80.5 %). The iCT-nav group had the lowest revision rate due to loosening (p < 0.001), while the FG group showed the highest revision rates due to misplacement (p < 0.001) and loosening (p = 0.001). Radiation exposure (effective dose, ED) was significantly lower in the iCT group compared to the FG group.

**Conclusion:**

RG PS placement demonstrates superior accuracy compared to iCT-nav and FG. Furthermore, intraoperative CT imaging significantly reduces total radiation exposure for patients.

## Highlights

• Robot-guided screws showed the highest accuracy in our cohort, with no postoperative revisions observed.
• Screw loosening varied across techniques; smaller pedicle and screw diameters were associated with higher risk.
• iCT-based approaches were linked to lower in-hospital radiation exposure than fluoroscopy.
• Image-guided techniques (RG and iCT-nav) showed favorable accuracy and radiation profiles compared with FG.

## 1 Introduction

Pedicle screw (PS) placement is a common surgical technique used to stabilize the spine in cases of instability due to degenerative, oncological, traumatic, and infectious diseases. Despite its widespread use across various methods—including freehand, fluoroscopy-guided (FG), and navigated techniques using either a navigated drill-guide or robotic arm—misplacement rates remain relatively high (Cammarata et al., 2023). A systematic review reported an overall revision rate of 2.9 % due to misplaced PS (Fichtner et al., 2018), with FG showing lower rates than freehand techniques and navigated or robot-guided (RG) approaches demonstrating improved outcomes compared to FG (Molliqaj et al., 2017; Budu et al., 2020).

Emerging literature supports the enhanced accuracy of spinal navigation and intraoperative CT (iCT) imaging over FG and freehand methods (Perdomo-Pantoja et al., 2019). However, high costs, technical limitations, and patient radiation exposure remain barriers to widespread adoption (Chatelain et al., 2023). While perioperative outcomes, such as revision rates and new neurological deficits, are reportedly similar across techniques, systematic reviews suggest that RG systems offer the highest accuracy, albeit with increased operative times (Al-Naseem et al., 2024; Riewruja et al., 2024).

This study aims to compare the accuracy and revision rates of RG, iCT-nav, and FG PS placement in a single-center setting, assessing screw loosening and technique-specific outcomes across different spinal pathologies. Notably, RG was introduced in our department only 14 months after the study period began. We hypothesized that RG would achieve superior outcomes, despite the team's initial learning curve. “We hypothesized that robot-guided pedicle-screw placement achieves higher accuracy and lower revision rates compared with intraoperative CT-navigation and fluoroscopy-guided techniques in a single-center, two-surgeon setting.” This was our main hypothesis. We also anticipated that low-dose intraoperative CT would reduce overall radiation exposure compared to FG.

## 2 Materials and methods

We conducted a retrospective single-center study, analyzing data from 237 consecutive patients who underwent titanium PS placement using RG, iCT-nav, or FG techniques between January 2020 and June 2023. All procedures followed the 1964 Declaration of Helsinki, and written informed consent was obtained from all patients. The Ethics Committee of University Hospital XX waived additional approval due to the study's retrospective design (Az: RS 22/72).

Surgeries were performed by two spinal surgeons (first/corresponding and senior authors) in patients with degenerative, traumatic, infectious, or oncologic spinal conditions. All 237 cases were performed by two trained spinal neurosurgeons (first and senior authors). The study observation period began in March 2021, corresponding to approximately 5 and 3 years of independent surgical experience after completion of residency, respectively, at the beginning of data collection. Both had extensive experience with fluoroscopy-guided and iCT-navigated procedures prior to this study. The robotic system was introduced institutionally in March 2021 and implemented jointly by these two surgeons after formal training.

FG placement used standard intraoperative C-arm imaging, while navigation relied on the BrainLab platform with iCT. The robotic arm (CIRQ®, BrainLab) was used in all RG cases. RG surgeries also employed iCT for registration and implant position verification; the first 13 RG cases used fluoroscopy-assisted screw placement, while later cases transitioned to fully navigated screws.

In iCT-nav procedures, iCT was used for automatic registration and navigated K-wire and screw placement. For open procedures, the reference array was fixed to the exposed spinous process at a level adjacent and proximal to the first instrumented segment. In percutaneous cases, it was inserted through a small stab incision at the same relative position. In long-segment constructs or instability cases, the array was repositioned intraoperatively to a level adjacent to the most caudal or central instrumented segment, followed by a repeated registration scan. The workflow was standardized and applied identically in both open and percutaneous approaches. FG cases relied entirely on fluoroscopy. Surgical technique selection depended on iCT availability, as only one operating room was iCT-equipped. Due to instrument limitations, only one RG case could be performed per day. RG was introduced in March 2021. Follow-up continued until December 2024.

Clinical data, including neurological status, BMI, osteoporosis, diagnosis, and technique, were extracted from patient records. Screw and pedicle measurements were taken from surgical protocols and CT scans. Pedicle width was measured at the axial midpoint by the first and third authors, with discrepancies resolved by the senior author (Abbas et al., 2020; Omar Pacha et al., 2020).

Each PS was classified using the Gertzbein-Robbins Scale (GRS), Zdichavsky, and Heary classification systems. Classifications were conducted independently by the first and third authors using anonymized CT data. Disagreements were resolved by the senior author. Intraoperative CT was used in all RG and iCT-nav cases; postoperative CT was performed in all FG cases. Additional CTs were performed as needed.

GRS classification classifies PS placement according to following criteria (Gertzbein and Robbins, 1990):

Grade A: The screw is fully contained within the pedicle.

Grade B: The screw breaches the pedicle cortex by less than 2 mm.

Grade C: The screw breaches the pedicle cortex by 2–4 mm.

Grade D: The screw breaches the pedicle cortex by 4–6 mm.

Grade E: The screw breaches the pedicle cortex by 6 mm or more, or is completely outside the pedicle.

Zdichavsky grading system (Theologou et al., 2017; Zdichavsky et al., 2004):

IA: ≥50 % of pedicle screw diameter (PSD) within the pedicle & ≥ 50 % of PSD within the vertebral body.

IB: >50 % of PSD lateral outside the pedicle & > 50 % of PSD within the vertebral body.

IIA: ≥50 % of PSD within the pedicle & > 50 % of PSD lateral outside the vertebral body.

IIB: ≥50 % of PSD within the pedicle & tip of PS crossing the middle line of the vertebral body.

IIIA: >50 % of PSD lateral outside the pedicle & >50 % of PSD lateral outside the vertebral body.

IIIB:>50 % of PSD medial outside the pedicle & tip of PS crossing midline of the vertebral body.

The Heary classification (Heary et al., 2004):

Grade I: entirely contained within pedicle.

Grade II:, violates lateral pedicle but screw tip entirely contained within the vertebral body (VB);

Grade III: tip penetrates anterior or lateral VB;

Grade IV: breaches medial or inferior pedicle;

Grade V: violates pedicle or VB and endangers spinal cord, nerve root, or great vessels and requires immediate revision.

The classification of the screws was independently performed by the first and third authors. Both authors conducted the assessment without knowledge of each other's results or the patients' medical histories, using anonymized intraoperative CT (iCT) or postoperative CT data. Anonymization was achieved through patient identification numbers from our hospital system. In cases of disagreement, the final classification was determined by the senior author.

In both the iCT-navigation (iCT-nav) and robotic-guided (RG) groups, intraoperative CT was performed in all cases. In the fluoroscopy-guided (FG) group, postoperative CT was routinely conducted on the day following surgery. Additional spinal CT examinations were carried out when further surgical procedures were necessary, such as screw revision or to verify implant positioning following vertebral body implant insertion.

The surgical workflow involving intraoperative imaging with a mobile 32-slice CT scanner (AIRO®, Brainlab, Munich, Germany), which is closely integrated into a navigation system, has been previously described for iCT-nav and robotic cases (Carl et al., 2019; Pojskić et al., 2021). Briefly, in RG and iCT cases, an iCT registration scan was obtained either after exposure of the target region and fixation of the reference array to the proximal spinous process in open procedures, or at the beginning of the operation in percutaneous cases via a small incision for reference array placement.

In iCT cases, pedicle screws (PS) were inserted using a navigated drill guide and navigated screws. In the first 13 RG cases, PS placement followed RG-assisted K-wire insertion and was completed under fluoroscopic guidance. In subsequent cases, PS were placed with navigation. After screw placement, an intraoperative control CT scan was performed to assess the positioning of the screws. If suboptimal positioning was detected, screw revision or removal was conducted, followed by another iCT scan to confirm final implant position.

Suboptimal PS positioning was defined as screws graded D or E according to the Gertzbein-Robbins Scale (GRS). D-grade screws located centrally within the construct were either removed or left in place, depending on clinical judgment, whereas D-grade screws at the ends of the construct were revised due to potential stability concerns. In the FG group, indications for screw revision were based on postoperative CT using the same criteria.

Cage implantation and final X-ray imaging of the spinal construct were performed under fluoroscopic guidance in all cases. Postoperative CT follow-up was performed as part of routine clinical care. Radiological screw loosening was defined as the presence of a radiolucent rim >1 mm around at least one screw on X-ray or CT, without corresponding clinical symptoms (Marie-Hardy et al., 2020; Sandén et al., 2004; Abul-Kasim and Ohlin, 2014). Symptomatic screw loosening was defined as loosening associated with pain and spinal instability.

Radiation exposure for staff and patients was measured based on cumulative radiation doses associated with surgical treatment of the spine. Radiation exposure for operating room staff was assessed using the documented dose-area product (DAP) from the fluoroscopy unit as a surrogate marker, as personal dosimeter measurements were not available for all procedures. Because staff leaves the room during intraoperative CT acquisition, reported iCT-related radiation values reflect patient dose only, while staff exposure was effectively negligible. In FG cases, the dose area product (DAP) and the fluoroscopy time were documented from the X-ray protocols (Carl et al., 2019). In addition, the overall total dose length product (DLP), DLP per scan and per scout length, the selected CT protocol, and the current intensity (amperage) were documented for the iCT-nav and RG group (Carl et al., 2019). The calculation of the effective dose (ED) was based on current ED/DLP conversion factors, which are estimated at 5.4 μSv/Gy·cm^2^ for cervical spine examinations, 17.8 μSv/Gy·cm^2^ for the thoracic spine and 19.8 μSv/Gy·cm^2^ for the lumbar spine (Huda et al., 2008; Elbakri and Kirkpatrick, 2013). In addition, the ED from postoperative CT scans performed to check the position of implants (screws, expandable cage after corpectomy) were added to the cumulative dose.

Statistical analysis was conducted using SPSS (v27.0). Continuous variables were expressed as means or medians with 95 % confidence intervals; categorical variables as frequencies and percentages. The Kolmogorov-Smirnov test was used to evaluate the normality of the distributions. As the data were not normally distributed, non-parametric methods were applied. Statistically significant differences among categorical variables were assessed using Pearson's Chi-square test, while differences among continuous variables were evaluated with the Kruskal-Wallis H test. Comparisons of total effective dose (ED) between iCT cases (including both RG and navigation approaches) and the FG technique were performed using the Mann-Whitney *U* test. Statistical significance was defined as p ≤ 0.05.Statistical significance was defined as p ≤ 0.05.

## 3 Results

### 3.1 General characteristics of the patients and neurological outcome

A total of 237 patients (mean age 68.7 ± 12.7 years, 52.3 % female) underwent instrumentation surgery with 1352 PS placements by two experienced spine surgeons. The most common indications were degenerative disorders (33.8 %), trauma (25.3 %), tumors (19.8 %), and infections (21.1 %).

Among the 237 patients included, 46 (19.4 %) underwent CT-navigated instrumentation, 120 (50.6 %) were treated with fluoroscopy guidance, and 71 (30.0 %) with robotic assistance. Preoperatively, 107 patients (45.1 %) presented with neurological deficits. Postoperatively, neurological deficits were observed in 88 patients (37.1 %).

Functional outcomes at follow-up demonstrated that 143 patients (60.3 %) remained neurologically unchanged. Of these, 131 patients (55.3 %) had no neurological deficit pre- and postoperatively, while 12 patients (5.1 %) exhibited persistent deficits without change. Eighteen patients (7.6 %) experienced complete recovery of preoperative neurological symptoms. Partial improvement, defined as reduction but not complete resolution of neurological deficits, was achieved in 70 patients (29.5 %). Neurological deterioration was documented in 6 patients (2.5 %). Deterioration was not due to screw malposition.

In CT-navigation group preoperatively, 21 patients (45.7 %) presented with neurological deficits. Postoperatively, 17 patients (37.0 %) exhibited deficits. Regarding functional outcomes, 28 patients (60.9 %) remained unchanged, including 25 patients (54.3 %) without neurological deficits and 3 patients (6.5 %) with persistent deficits. Complete recovery was observed in 3 patients (6.5 %), partial improvement in 13 patients (28.3 %), and neurological deterioration in 2 patients (4.3 %).

Of 120 patients in FG group, 54 patients (45.0 %) had neurological deficits preoperatively, and 44 patients (36.7 %) remained with deficits postoperatively. In terms of outcomes, 73 patients (60.8 %) were unchanged, including 66 patients (55.0 %) without deficits and 7 patients (5.8 %) with persistent deficits. Full recovery occurred in 9 patients (7.5 %), partial improvement in 34 patients (28.3 %), while 4 patients (3.3 %) deteriorated.

In RG group, preoperative neurological deficits were present in 32 patients (45.1 %), and postoperative deficits in 27 patients (38.0 %). Functionally, 42 patients (59.2 %) remained unchanged, including 40 patients (56.3 %) without neurological deficits and 2 patients (2.8 %) with unchanged deficits. Five patients (7.0 %) achieved full recovery, 23 patients (32.4 %) improved with residual deficits, and deterioration occurred in 0 patients (0 %).

### 3.2 PS placement techniques

Three techniques were used: FG, iCT-nav, and RG. The choice of technique depended primarily on iCT availability. Screws were placed at the following spinal levels: cervical (3.4 %), lumbar (46.9 %), thoracic (44.8 %), and sacral (4.9 %).

Of the 237 surgeries, the first author performed 126 cases (740 screws, 54.7 %), and the senior author performed 111 cases (612 screws, 45.3 %). In 13 % of cases, both surgeons were involved.

There were no statistically significant differences in BMI, osteoporosis, underlying pathology, surgical approach (open vs. percutaneous), pedicle diameter, or screw-to-pedicle diameter ratios among the three technique groups (p > 0.05).

Table 1 summarizes characteristics of the entire cohort with baseline demographic and procedure characteristics.

**Table 1:** Patient characteristics (N = 237).

Variable	N	%
**Sex**		
Female	113	47.7
**Age**		
>71 years old	112	47.3
**Diagnosis**		
Degenerative	80	33.8
Fracture	60	25.3
Tumor (primary or metastasis)	47	19.8
Spondylodiscitis	50	21.1
**Previous non-instrumented spine surgery (fused levels)**	82	34.6
**Surgical Technique (open/percutaneous)**		
Open	191	80.6
Percutaneous	46	19.4
**Surgical Technique (iCT-nav, FG, RG)**		
iCT-navigation	46	19.4
Fluoroscopy-guided (FG)	120	50.6
Robotic-guided (RG)	71	30.0
**Neurological Deficit**		
Preoperative	107	45.1
Postoperative	88	37.1
**Spine Section** (C = cervical, Th = thoracic, L = lumbar, S = sacral)		
CS/CS	4	1.7
CS/ThS	15	6.3
LS/LS	81	34.2
LS/SS	31	13.1
ThS/LS	43	18.1
ThS/SS	1	0.4
ThS/ThS	62	26.2
**Additional decompression in same surgery**		
Yes	161	67.9
No	76	32.1
**Obesity (BMI > 30)**	61	25.7
**Osteoporosis**	39	16.5
**Screw Revision**		
Due to postoperative misplacement	14	5.9
Due to loosening	9	3.8
Due to intraoperative misplacement	10	4.2

### 3.3 Complications

Neurological complications apart from screw misplacement included wound healing deficits and cerebrospinal fluid (CSF) leakage in 30 patients (12.7 % overall), with comparable rates across groups: iCT-nav 6 patients (13.0 %), FG 15 patients (12.5 %), and RG 9 patients (12.7 %) (p = 0.98). Systemic complications such as septic shock, pulmonary embolism, and pleural effusion occurred in 18 patients (7.6 % overall), distributed as follows: iCT-nav 8.7 %, FG 7.5 %, and RG 7.0 % (p = 0.94). Screw malposition did not result in any neurological injury or cerebrospinal fluid (CSF) leak in any case. In-hospital mortality was recorded in 10 patients (4.2 % overall), again without relevant differences between groups: iCT-nav 4.3 %, FG 4.2 %, and RG 4.2 % (p = 0.524).

### 3.4 Mean hospital stay

Mean hospital stay was 14.2 ± 10 days and did not differ significantly among the three techniques. However, patients with trauma, tumor, or infection had longer stays than those with degenerative conditions (p > 0.05).

### 3.5 Mean operative time

Mean operative time was 232.5 ± 116.4 min overall. Only the total operative time was documented; screw placement time was not recorded separately FG surgeries averaged 214.9 ± 110.8 min, iCT-nav 273.9 ± 126.5 min, and RG 241.9 ± 118.4 min iCT-related techniques had significantly longer operative times than FG (p < 0.05), though differences between RG and iCT-nav were not significant.

### 3.6 RG PS placement shows increased overall accuracy and accuracy of “perfect” GRS A screws compared to iCT-nav and FG PS

Screw distribution, classification and revision rates (Supplementary Table 2.) showed statistically significant differences among the three techniques for all classification systems (GRS, Zdichavsky, Heary). RG demonstrated the highest PS accuracy (GRS A: 91.7 %), followed by iCT-nav (86.2 %) and FG (80.5 %). No postoperative screw revisions were required in the RG group.

### 3.7 Screw revisions

Seventeen screws (1.3 %) were revised intraoperatively after misplacement was detected on CT, and further 22 screws (1.6 %) were revised postoperatively, resulting in an overall correction rate due to misplacement of 2.9 % (39 screws).

Of 1352 screws, 25 screws were GRS D and E (1.9 %). No new neurological deficits occurred due to screw misplacement. A total of 5 GRS E screws (0.38 %) showed a medial breach. A total of 2 screws that were revised intraoperatively had a GRS E with medial breach; this patient had no new neurological deficits. Of 22 screws revised postoperatively, only 3 screws were GRS E screws with medial breach that caused pain, but no new neurological deficits. Overall, thirteen cases (5.5 %) underwent revision surgery to correct the screws postoperatively, and in 10 cases (4.2 %) the revision was performed intraoperatively.

A total of 52 screws (3.8 %) were revised intra- or postoperatively. The postoperative revision rate due to misplacement was 0 % in the RG group, 1.3 % in the iCT-nav group, and 4.2 % in the FG group (p < 0.001). Revisions due to loosening occurred in 0 %, 0.8 %, and 2.3 %, respectively (p = 0.001). The robotic group required no postoperative revisions but had ten intraoperative corrections after intraoperative CT control (Table 2).

**Table 2:** Revision rates and timing per technique.

Technique	Total screws (n)	Intra-op revisions n (%)	Post-op revisions n (%)	Due to misplacement n (%)	Due to loosening n (%)	Total revised n (%)
Robotic-guided (RG)	444	10 (2.3 %)	0 (0 %)	10 (2.3 %)	0 (0 %)	10 (2.3 %)
iCT-navigation (iCT-nav)	241	7 (2.9 %)	5 (2.1 %)	3 (1.3 %)	2 (0.8 %)	12 (5.0 %)
Fluoroscopy-guided (FG)	667	0 (0 %)	43 (6.5 %)	28 (4.2 %)	15 (2.3 %)	43 (6.5 %)
**Total**	**1352**	**17 (1.3 %)**	**48 (3.6 %)**	**41 (3.0 %)**	**17 (1.3 %)**	**52 (3.8 %)**

### 3.8 Pedicle screw (PS) loosening was least frequent in the iCT-navigation (iCT-nav) group and most frequent in the fluoroscopy-guided (FG) group

Postoperative CT follow-up was performed as part of routine clinical care. at a median of 8 months (IQR = 5–17 months) to assess fusion and screw loosening. In total, 87 screws demonstrated radiological loosening across 31 patients during the follow up, whereas 30 screws (2.2 %) in 9 patients required revision surgery; the remaining cases represented asymptomatic or clinically stable loosening that did not necessitate operative management. In the subgroup of patients in whom screw loosening occurred, the mean follow-up interval was 9.8 ± 6.9 months. Among these 31 patients, loosening occurred in 14 fusion constructs and 17 instrumentation-only procedures. No cases of screw pull-out or breakage were observed. The lowest revision rate for screw loosening was identified in the iCT-nav group (p < 0.001). Conversely, the FG group demonstrated the highest revision rates both for screw misplacement (p < 0.001) and screw loosening (p = 0.001).

### 3.9 Factors influencing pedicle screw placement accuracy

Indication for surgery (degenerative disease, tumor, fracture, or infection), clinical outcome (improved, unchanged, or worsened neurological status), laterality of PS placement (left vs. right), spinal region (cervical, thoracic, lumbar, sacral, or junctional), body mass index (BMI), osteoporosis, and surgical approach (open vs. percutaneous) did not significantly affect PS placement accuracy (p > 0.05). Furthermore, no significant differences were found between the two operating surgeons with respect to the distribution of techniques (RG, iCT-nav, FG), PS placement accuracy rates, frequency of GRS A screw placement, or intraoperative and postoperative revision rates (p > 0.05).

### 3.10 Screw and pedicle diameter as predictors of long-term screw loosening

Table 3 presents the analysis of long-term screw loosening. Both screw diameter (AUC 0.608, cut-off ≤6.5 mm) and pedicle diameter (AUC 0.612, cut-off ≤12 mm) emerged as significant predictors, each demonstrating high sensitivity (85.38 % and 83.19 %, respectively) but low specificity (31.03 % and 35.63 %, respectively; p < 0.001). These findings suggest that smaller screw and pedicle diameters are associated with an increased risk of screw loosening, albeit at the cost of a high false-positive rate.

**Table 3:** Long-course screw loosening.

Variable	AUC	95 % CI	Cut-off		Sensitivity	Specificity	p
Screw diameter (mm)	0.608	0.581–0.634	≤6.5		85.38	31.03	<0.001
Pedicle diameter (mm)	0.612	0.586–0.638	≤12		83.19	35.63	<0.001

A total of 1140 screws (84.3 %) had a diameter of ≤6.5 mm. The mean pedicle diameter was 19.9 ± 2.5 mm in the sacral spine, 11.23 ± 9.0 mm in the lumbar spine, 7.65 ± 1.5 mm in the thoracic spine, and 6.6 ± 1.2 mm in the cervical spine.

Morphometric risk factors for screw loosening are summarized in Table 4. A pedicle diameter of ≤12 mm was associated with a 2.74-fold increased risk of screw loosening (OR = 2.7391, 95 % CI: 1.7241–4.3517, p < 0.001). Similarly, a screw diameter of ≤6.5 mm was associated with a 2.63-fold increased risk (OR = 2.6270, 95 % CI: 1.6252–4.2465, p < 0.001).

**Table 4:** Morphometric predictors of screw loosening.

Variable	Odds ratio	95 % CI	p
Pedicle diameter ≤12 mm	2.7391	1.7241–4.3517	<0.001
Screw diameter ≤6.5	2.6270	1.6252–4.2465	<0.001

### 3.11 The total radiation exposure is lower in iCT cases compared to the FG group

Mean DLP from intraoperative imaging was: FG 879.3 ± 648.4 cGy cm^2^, iCT 169.7 ± 202.3 cGy cm^2^. The iCT group had significantly lower radiation exposure than FG (p < 0.01). In 19 FG and 12 iCT cases, additional CTs were performed for implant verification or revision.

**Fig. 1:**
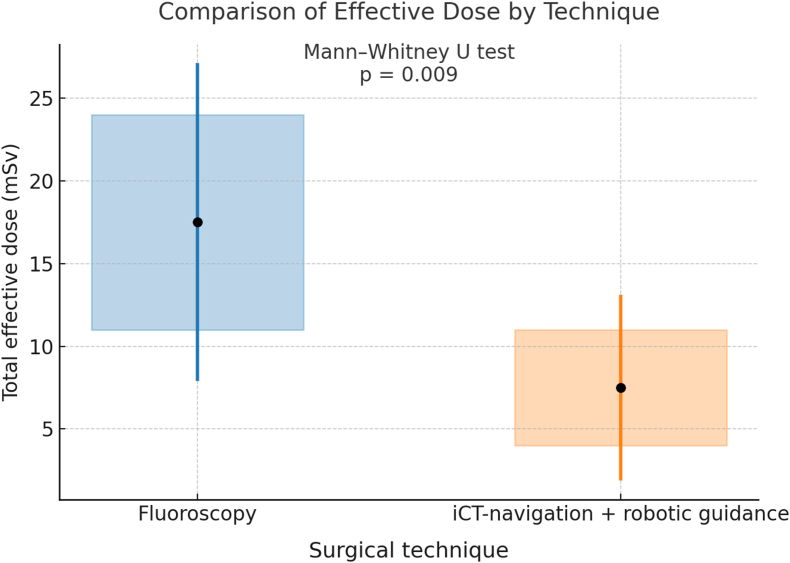
Comparison of total effective dose (ED) in iCT cases (RG and navigation) compared to FG technique.

The mean total effective radiation dose was lower in iCT cases (7.4 ± 5.2 mSv) compared to FG cases (17.4 ± 9.5 mSv), including intra- and postoperative scans. This difference was statistically significant ((p = 0.009) (Fig. 1).

## 4 Discussion

### 4.1 Principal findings and study novelty

In this single-center retrospective analysis, we RG, iCT-nav, and FG PS placement performed by two trained spine surgeons. The primary endpoint was PS accuracy, while secondary endpoints included revision rate, screw loosening and radiation exposure. Among 1352 screws, accuracy differed significantly between modalities, with RG demonstrating 91.7 % Grade A placement, iCT-nav 86.2 %, and FG 80.5 %. These values fall within the reported ranges of prior image-guided accuracy studies (Fatima et al., 2021; Naik et al., 2022), and the lower accuracy observed with FG is consistent with published evidence describing higher malposition rates under conventional fluoroscopy (Molliqaj et al., 2017). Fifty-two screws (3.8 %) required revision overall; while none of the RG screws were revised postoperatively, ten RG screws (2.3 %) were corrected intraoperatively following CT verification, aligning with reports that intraoperative imaging facilitates immediate correction and prevents delayed revision. Screw loosening was identified in 6.4 % of cases, with pedicle diameter ≤12 mm and screw size ≤6.5 mm recognized as risk factors in earlier studies, while BMI, osteoporosis, indication, and approach did not significantly influence accuracy, consistent with recent findings (Marie-Hardy et al., 2020; Wu et al., 2025). Operative time was longest in the iCT-nav and RG cohorts, reflecting additional imaging, calibration, and registration steps, a trend comparable to previous workflow analyses (Riewruja et al., 2024).

This study demonstrated measurable differences in pedicle screw placement accuracy and revision rates across RG, iCT-nav, and FG techniques. RG showed the highest proportion of GRS A screws and no postoperative screw revisions, reflecting the accuracy achieved within our institutional workflow, even during the early implementation phase. The dataset benefits from single-center consistency, the involvement of two experienced surgeons, and the analysis of more than 1300 screws. Although retrospective in design, these observations illustrate how each technique performed in our clinical setting without permitting conclusions regarding procedural superiority. By reporting cumulative radiation dose—including both intraoperative and postoperative imaging—we also provide descriptive insight into radiation exposure patterns, with iCT-based navigation demonstrating lower total exposure within the parameters of our cohort. Radiation exposure differed between techniques, with cumulative effective dose significantly lower in iCT-based workflows compared with FG, demonstrating that low-dose intraoperative CT can reduce total radiation exposure. As these outcomes derive from a retrospective, non-randomized dataset, they should be interpreted descriptively. No conclusions favoring one modality over another can be drawn, and the results primarily reflect technique-specific workflow characteristics within our institutional setting.

### 4.2 Screw placement accuracy

Previous studies confirm the superiority of navigated and RG screw placement over freehand and FG techniques (Cammarata et al., 2023; Li et al., 2024). Meta-analyses have reported lower rates of cortical breaches and higher accuracy with RG systems. Robotic guidance (RG) has been shown to be superior to freehand pedicle screw (PS) placement, as demonstrated by systematic reviews encompassing 7379 screws across 19 studies conducted between 1980 and 2020 (Fatima et al., 2021). This superiority has been observed in the context of posterior spinal surgery for scoliosis, as well as in single-center studies involving mixed patient cohorts with various surgical indications, where reported accuracy rates ranged from 95 % to 99 % (Al-Naseem et al., 2024; Li et al., 2024; Wang et al., 2024), as (Asada et al., 2024a; Farber et al., 2021; Tsai et al., 2016; Zhang et al., 2021; Gabrovsky et al., 2023; Matur et al., 2023). Notably, RG has demonstrated comparable accuracy in both open and percutaneous approaches (Fan et al., 2022). Furthermore, several studies have reported that RG provides higher accuracy in PS placement than other navigation methods, including those using C-arm, O-arm, or intraoperative CT navigation (iCT-nav) systems (Naik et al., 2022; Yu et al., 2023; Romagna et al., 2023; Shahi et al., 2022). Systematic reviews have also indicated a reduction in overall complication rates when RG is used, compared to other techniques (Naik et al., 2011). An exception is the review by Perdomo-Pantoja et al., which reported higher PS placement accuracy in the iCT-nav group overall, although RG was associated with a lower rate of medial breaches in the thoracic spine (Perdomo-Pantoja et al., 2019).

Since RG was introduced only recently in our department, both treating surgeons were still early in their experience with this technique. Nevertheless, RG achieved accuracy values comparable to or numerically higher than our established FG and iCT-nav workflows within this study period. Our findings are consistent with reports describing high placement precision with robotic systems, including in anatomically demanding cases, but given the retrospective design and heterogeneous case mix, these observations should be interpreted descriptively.

### 4.3 Revision rates and misplacement

Consistent with literature, the FG group exhibited the highest screw revision rate (Molliqaj et al., 2017). Reported misplacement rates vary widely (from 0.3 % (Chatelain et al., 2023), to 6.6 % (Molliqaj et al., 2017)) due to heterogeneity in definitions, assessment tools, and the lack of routine postoperative CT. In our study, revisions were based on CT-confirmed malpositions, minimizing underreporting. Notably, none of the GRS E medial breaches resulted in new neurological deficits. While RG minimizes certain error sources seen in navigation—such as registration drift or misalignment due to distance from the reference array—errors can still occur due to skiving or mechanical interference. Understanding these failure points can guide future refinements (Jin et al., 2017; Gautam et al., 2024).

### 4.4 Differences between RG, iCT-nav and FG technique: operative time, proximal facet joint violation, screw dimensions and complication rate

RG and iCT-nav cases required longer operative times compared to FG, consistent with reports citing additional setup, calibration, and scan durations (Al-Naseem et al., 2024; Guan et al., 2024). However, this increase was offset by reduced screw revision rates and improved accuracy. The premise is that use of iCT prolongs the operating time due to installation of the robotic arm, the calibration of the instruments for navigation, and the iCT scans. Operative time is influenced by multiple factors beyond screw placement—including exposure, decompression, fusion length, and additional procedures—thus limiting its utility as a comparative metric. Future prospective trials should isolate and quantify the duration of screw placement alone. The increase in operative time associated with robotic guidance (RG) appears justified when considering the reduced overall radiation exposure to both patients and operating room staff, improved pedicle screw (PS) placement accuracy, and lower revision rates.

The superiority of RG over the freehand (FH) technique is evident in its higher overall accuracy, decreased rates of screw revision, and reduced incidence of proximal facet joint violations (Guan et al., 2024; Zhang et al., 2022). The clinical benefits of RG were further supported by a retrospective study demonstrating reduced intraoperative blood loss and shorter hospital stays in patients undergoing primary lumbar fusion surgery, without an increase in complication rates (Asada et al., 2024b). However, these benefits were not observed in our cohort, potentially due to the limited number of less invasive percutaneous procedures and the high proportion of patients requiring prolonged hospitalization owing to the severity of underlying oncologic or infectious pathologies, as well as healthcare system-specific factors.

RG has also been associated with a lower rate of proximal facet joint violations and facilitates the placement of larger-diameter screws, which may contribute to enhanced construct stability and improved fusion outcomes. While differences in clinical outcomes between RG and intraoperative CT navigation (iCT-nav) appear minimal (Mao et al., 2020), some studies suggest that RG offers marginally superior PS accuracy, allows for the insertion of larger screws, and reduces radiation exposure (Shahi et al., 2022; Shafi et al., 2022).

### 4.5 PS loosening

Screw loosening is a common problem after stabilization surgery, which does not always cause symptoms or necessitates revision. Our study has shown a total screw loosening rate of 6.4 %, with 2.2.% or 30 screws which required revision during the follow up. The causes are varied and range from screw loosening due to complex suboptimal biomechanical relationships between bone quality, pedicle diameter, screw diameter and length, to instability of the spine in connection with diseases of adjacent segments, to loosening after successful fusion and bony bridging. Our data show that pedicle diameter of ≤12 mm and a screw diameter of ≤6.5 mm was significantly associated with higher odds of screw loosening, and that revision rate due to loosening was highest in FG group. Pedicle screw diameter tends to be larger in males than females across all lumbar levels, as well as in patients with degenerative stenosis of the spinal canal, whereas age and BMI do not seem to show an effect to pedicle width (Abbas et al., 2020). At 2 years following surgery in 81 patients with adolescent idiopathic scoliosis (AIS), 32 % of patients have shown signs of loosening, predominantly male, with only 5 patients who experienced pain and one patient who underwent revision due to new deficit caused by the loosened screw (Abul-Kasim and Ohlin, 2014). In cohorts comparing RG and FG PS placement, RG technique tends to yield placement of PS screws of significantly larger diameter with not significant, but lower loosening rate (Du et al., 2022; Lai et al., 2022). Some potential risk factors for screw loosening include length of spinal construct, age of the patient, distance from the upper endplate and the screw (Lai et al., 2022), scoliosis, sagittal imbalance and osteoporosis (Marie-Hardy et al., 2020).

### 4.6 Cost-effectiveness of RG PS placement

Although we did not conduct a formal cost-effectiveness analysis, published data suggest that RG can reduce revision surgeries and length of stay, partially offsetting the initial investment in equipment and training (Menger et al., 2018; Sturgill et al., 2024). Reimbursement models differ by country, and the lack of uniform billing practices (e.g., in Germany's DRG system) complicates such analyses. Still, increased use of minimally invasive techniques enabled by RG and decreased complication rates support the long-term economic value of robotic assistance.

A recent systematic review found that, while similar outcomes were observed for lumbar fusion across robotic-guided (RG), intraoperative CT navigation (iCT-nav), and fluoroscopy-guided (FG) techniques, RG was associated with a reduced length of hospital stay and, indirectly, with cost savings (Sturgill et al., 2024). In the present study, the cost-effectiveness of RG for pedicle screw (PS) placement was not assessed due to limitations imposed by the national healthcare reimbursement system. Specifically, differences between public and private insurance schemes and the heterogeneity of the available data precluded a reliable cost-effectiveness analysis.

Several factors influence the overall cost-effectiveness of RG, including the initial investment in the robotic system, training requirements, operative time, revision rates, and length of hospitalization (Haik et al., 2025). An analysis of a national spinal surgery database in the United States concluded that RG is cost-effective, as it enables safer use of minimally invasive techniques over open surgery, reduces operative duration and hospital stays, and lowers the incidence of postoperative infections (Menger et al., 2018). However, other studies have reported higher index surgery costs for RG, primarily attributable to increased supply and operating room expenses (Ezeokoli et al., 2022). Importantly, these studies focused solely on the index procedure, without accounting for potential cost savings associated with reduced revision surgery rates, which are a critical component of overall cost-effectiveness.

In elective thoracolumbar stabilization procedures, the enhanced accuracy of RG-assisted PS placement was estimated to prevent approximately 9.47 revision surgeries per cohort (Menger et al., 2018). Critics of RG, particularly proponents of iCT-navigation, have referred to the robotic arm as a “million-dollar drill guide”, highlighting the perceived cost disadvantage of RG compared to conventional navigation methods (D'Souza et al., 2019). However, the robotic arm provides increased mechanical rigidity and stability during instrumentation, eliminating the need for an additional assistant to stabilize the navigated drill guide. This enhanced stability likely contributes to improved accuracy in K-wire and screw placement. Such stability is particularly crucial in open procedures following prior decompression, where any inadvertent movement or obstruction of the instrument holder near the spinal cord or nerve roots could result in catastrophic injury.

### 4.7 Radiation exposure

Our findings confirm that iCT reduces total radiation exposure when low-dose protocols are used and postoperative scans are avoided. Many prior studies focused only on intraoperative radiation, overlooking postoperative imaging, which can significantly inflate total exposure. By incorporating all scans into cumulative ED calculations, our study offers a more accurate comparison and highlights a key advantage of intraoperative CT navigation: reduced staff and patient radiation without compromising surgical accuracy.

Intraoperative CT (iCT) has been shown to minimize radiation exposure to surgical staff compared to fluoroscopy-guided (FG) techniques, though at the cost of increased radiation exposure to the patient (Pennington et al., 2019). A recent study involving 156 patients and 918 pedicle screws placed in the thoracic and lumbar spine for various indications reported higher screw placement accuracy when intraoperative imaging was used. However, this was associated with a higher mean effective dose (ED) of radiation—9.69 mSv compared to 0.71 mSv for freehand fluoroscopy (Chatelain et al., 2023). Similarly, a retrospective study by Vadalà et al. also found elevated intraoperative radiation doses in iCT cases (Vadalà et al., 2024). Notably, in both studies, postoperative imaging was not included in the calculation of total patient ED, and radiation exposure to surgical staff was not addressed (Vadalà et al., 2024).

A systematic review of 38 studies, despite a low level of evidence and high risk of bias, concluded that FG using a conventional C-arm results in higher radiation exposure for both patients and staff compared to navigation-assisted or robotic-guided (RG) techniques employing C-arm or O-arm platforms (Caelers et al., 2023). This review, like others, focused primarily on intraoperative radiation exposure. For instance, a systematic review spanning 40 years of spinal stabilization surgeries reported that RG was associated with lower intraoperative radiation doses and longer operative times compared to freehand techniques (Fatima et al., 2021). Another systematic review comparing RG and freehand transforaminal lumbar interbody fusion (TLIF) procedures found no difference in fluoroscopy time between groups, but did report a lower total radiation dose in RG cases (Guan et al., 2024).

A multicenter trial evaluating minimally invasive RG versus FG in adult degenerative spine fixation surgeries demonstrated a 50.8 % reduction in total fluoroscopy time in the RG group (Jamshidi et al., 2021). Additionally, a retrospective cohort study of 278 patients undergoing one- or two-level TLIF found that RG resulted in lower intraoperative radiation exposure compared to conventional navigation (Shahi et al., 2022). In their systematic review, Siccoli et al. reported longer hospital stays and a higher complication rate in the FG group, but no significant differences in intraoperative radiation exposure when measured in seconds of fluoroscopy time (Siccoli et al., 2019). However, the authors highlighted the limited quality of evidence and inconsistent reporting of radiation dose across studies. They cautioned against overinterpretation of results, particularly as many studies failed to account for the effective dose from preoperative or intraoperative CT scans.

In our study, we observed that radiation exposure to operating room staff was minimized in iCT-guided procedures. In these cases, the C-arm was used only for initial level localization or, in a limited number of RG cases, for verification of cage placement or pedicle screw positioning following K-wire insertion. Total patient ED during hospitalization was significantly lower in the iCT group, primarily due to the use of low-dose protocols and the elimination of the need for postoperative CT imaging**.**

### 4.8 Limitations

Limitations include the retrospective design, single-center setting, and lack of randomization. The assignment of technique was influenced by room and equipment availability, potentially introducing selection bias. The clinical decision to use iCT was made in most cases for more challenging cases (such as for lower cervical and upper thoracic levels, which poses a risk of inclusion bias in the context of this study) and longer constructs, and the use of the RG-PS technique was initiated and primarily intended for FG-PS placement, with which both treating surgeons had experience. Nevertheless, baseline characteristics were balanced across groups.

The cost-effectiveness analysis of the use of RG-PS was not performed due to the specific reimbursement system in our country's complex diagnosis-related DRG system, as there are differences between public and private health insurance companies with regard to additional reimbursement for robot-assisted procedures, while the costs for intraoperative imaging such as iCT are reimbursed.

Furthermore, our results may not generalize to centers with different surgical experience or patient populations. Cost-effectiveness, blood loss, and proximal facet violation rates were not formally analyzed but should be addressed in future prospective trials. Despite these limitations, the large number of screws analyzed and consistent surgeon participation strengthen the internal validity of our findings.

## 5 Conclusion

In this cohort, robot-guided pedicle screw placement achieved the highest accuracy and the lowest observed postoperative revision rate within our institutional workflow, even during its early adoption phase. Intraoperative CT, particularly when used with low-dose protocols, contributed to lower cumulative radiation exposure compared with fluoroscopy-based procedures. These findings describe the performance characteristics of RG and iCT techniques in our setting and may inform their use in contemporary spine surgery, while recognizing that definitive conclusions regarding superiority or broad implementation cannot be drawn from this retrospective analysis.

## Declaration of competing interest

The authors declare the following financial interests/personal relationships which may be considered as potential competing interests: Christopher Nimsky, Miriam Bopp reports a relationship with Brainlab SE that includes: consulting or advisory. If there are other authors, they declare that they have no known competing financial interests or personal relationships that could have appeared to influence the work reported in this paper.
