# Supplementary material for: Comparative analysis of the pedicle screw accuracy, screw revision and loosening rate and radiation exposure of robotic-guided (RG), intraoperative computed tomography (iCT)-navigation guided, and fluoroscopy guided placement technique
Source: Brain Spine. 2025 Dec 4;6:105899. doi: 10.1016/j.bas.2025.105899 (PMC12756613; doi:10.1016/j.bas.2025.105899)
Supplement: Multimedia component 1 [file mmc1.docx]

**Table 2. Screw distribution, classification and revision rates**

| Category | iCT-nav (N = 241) | FG (N = 667) | RG (N = 444) | Total (N = 1352) | p* |
| --- | --- | --- | --- | --- | --- |
| Screw location |  |  |  |  | <0.001 |
| C3 | 0 (0.0) | 0 (0.0) | 2 (0.5) | 2 (0.1) |  |
| C6 | 4 (1.7) | 0 (0.0) | 6 (1.4) | 10 (0.7) |  |
| C7 | 28 (11.6) | 0 (0.0) | 6 (1.4) | 34 (2.5) |  |
| L1 | 4 (1.7) | 56 (8.4) | 34 (7.7) | 94 (7.0) |  |
| L2 | 2 (0.8) | 69 (10.3) | 48 (10.8) | 119 (8.8) |  |
| L3 | 5 (2.1) | 95 (14.2) | 45 (10.1) | 145 (10.7) |  |
| L4 | 13 (5.4) | 99 (14.8) | 33 (7.4) | 145 (10.7) |  |
| L5 | 15 (6.2) | 80 (12.0) | 36 (8.1) | 131 (9.7) |  |
| S1 | 6 (2.5) | 34 (5.1) | 24 (5.4) | 64 (4.7) |  |
| S2 | 2 (0.8) | 0 (0.0) | 0 (0.0) | 2 (0.1) |  |
| Th1 | 34 (14.1) | 12 (1.7) | 10 (2.3) | 56 (4.2) |  |
| Th10 | 6 (2.5) | 28 (4.2) | 24 (5.4) | 58 (4.3) |  |
| Th11 | 4 (1.7) | 40 (6.0) | 27 (6.1) | 71 (5.3) |  |
| Th12 | 0 (0.0) | 36 (5.4) | 21 (4.7) | 57 (4.2) |  |
| Th2 | 37 (15.4) | 14 (2.1) | 7 (1.6) | 58 (4.3) |  |
| Th3 | 20 (8.3) | 14 (2.1) | 9 (2.0) | 43 (3.2) |  |
| Th4 | 14 (5.8) | 14 (2.1) | 17 (3.8) | 45 (3.3) |  |
| Th5 | 11 (4.6) | 8 (1.2) | 23 (5.2) | 42 (3.1) |  |
| Th6 | 10 (4.1) | 12 (1.8) | 12 (2.7) | 34 (2.5) |  |
| Th7 | 16 (6.6) | 18 (2.7) | 15 (3.4) | 49 (3.6) |  |
| Th8 | 6 (2.5) | 18 (2.7) | 26 (5.9) | 50 (3.7) |  |
| Th9 | 4 (1.7) | 20 (3.0) | 19 (4.3) | 43 (3.2) |  |
| Side |  |  |  |  | 0.072 |
| left | 122 (50.6) | 333 (49.9) | 220 (49.5) | 675 (49.9) |  |
| right | 119 (49.4) | 334 (50.1) | 224 (50.5) | 677 (50.1) |  |
| Technique |  |  |  |  | 0.965 |
| open | 241 (100.0) | 503 (75.4) | 372 (83.8) | 1116 (82.5) |  |
| percutaneous | 0 (0.0) | 164 (24.6) | 72 (16.2) | 236 (17.5) |  |
| GRS-Classification** |  |  |  |  | <0.001 |
| A | 194 (80.5) | 575 (86.2) | 407 (91.7) | 1176 (87.0) |  |
| B | 29 (12.0) | 53 (7.9) | 23 (5.2) | 105 (7.8) |  |
| C | 14 (5.8) | 22 (3.3) | 10 (2.3) | 46 (3.4) |  |
| D | 1 (0.4) | 12 (1.8) | 3 (0.7) | 16 (1.2) |  |
| E | 3 (1.2) | 5 (0.7) | 1 (0.2) | 9 (0.7) |  |
| Zdichavsky-classification |  |  |  |  | 0.002 |
| Ia | 219 (90.9) | 621 (93.1) | 428 (96.4) | 1268 (93.8) |  |
| Ib | 10 (4.1) | 6 (0.9) | 8 (1.8) | 24 (1.8) |  |
| IIa | 1 (0.4) | 10 (1.5) | 3 (0.7) | 14 (1.0) |  |
| IIb | 5 (2.1) | 7 (1.0) | 3 (0.7) | 15 (1.1) |  |
| IIIa | 3 (1.2) | 3 (0.4) | 1 (0.2) | 7 (0.5) |  |
| IIIb | 3 (1.2) | 20 (3.0) | 1 (0.2) | 24 (1.8) |  |
| Heary-classification |  |  |  |  | <0.001 |
| I | 192 (79.7) | 565 (84.7) | 404 (91.0) | 1161 (85.9) |  |
| II | 31 (12.9) | 30 (4.5) | 20 (4.5) | 81 (6.0) |  |
| III | 3 (1.2) | 11 (1.6) | 3 (0.7) | 17 (1.3) |  |
| IV | 13 (5.4) | 55 (8.2) | 16 (3.6) | 84 (6.2) |  |
| V | 2 (0.8) | 6 (0.9) | 1 (0.2) | 9 (0.7) |  |
| Screw revision post-op |  |  |  |  | <0.001 |
| No | 238 (98.8) | 648 (97.2) | 444 (100.0) | 1330 (98.4) |  |
| Yes | 3 (1.2) | 19 (2.8) | 0 (0.0) | 22 (1.6) |  |
| Screw revision intra-op |  |  |  |  | 0.218 |
| No | 236 (97.9) | 662 (99.3) | 437 (98.4) | 1335 (98.7) |  |
| Yes | 5 (2.1) | 5 (0.7) | 7 (1.6) | 17 (1.3) |  |
| Screw revision loosening |  |  |  |  | 0.001 |
| No | 241 (100.0) | 642 (96.3) | 439 (98.9) | 1322 (97.8) |  |
| Yes | 0 (0.0) | 25 (3.7) | 5 (1.1) | 30 (2.2) |  |
| Screw loosening (long-term) |  |  |  |  | 0.024 |
| No | 233 (96.7) | 613 (91.9) | 419 (94.4) | 1265 (93.6) |  |
| Yes | 8 (3.3) | 54 (8.1) | 25 (5.6) | 87 (6.4) |  |

*, Pearson’s chi square test

** Disagreements between the first and third author in the GRS classification of a screw occurred in 25 screws (1.9%) where the final classification was made by the first author. Inter-observer reliability is very high, with 98.15% raw agreement and an estimated Cohen's Kappa > 0.9. The first author is a senior spine surgeon (5 years' experience as a senior neurosurgeon), the third author is a medical student writing his MD thesis at the Department of Neurosurgery, and the senior author is a senior spine surgeon (7 years' experience as a senior neurosurgeon).
