# Supplementary material for: Comparative analysis of the pedicle screw accuracy, screw revision and loosening rate and radiation exposure of robotic-guided (RG), intraoperative computed tomography (iCT)-navigation guided, and fluoroscopy guided placement technique
Source: Brain Spine. 2025 Dec 4;6:105899. doi: 10.1016/j.bas.2025.105899 (PMC12756613; doi:10.1016/j.bas.2025.105899)
Supplement: Multimedia component 2 [file mmc2.docx]

**Declarations**

**Ethics approval and consent to participate.** This study was conducted in accordance with the principles of the 1964 Declaration of Helsinki. All patients gave their written consent for their data to be used for research and publication purposes. Ethical approval was waived by the local Ethics committee of university hospital Marburg, Philipps University, Az: RS 22/72, from 9th November 2022, in view of the retrospective nature of the study and all the procedures being performed were part of routine care.

**Funding.** No funding was received for conducting this study.

**Conflict of interest.** Ch. Nimsky and M. Bopp serve as scientific consultants to BrainLab. The author(s) declared no potential conflicts of interest with respect to the research, authorship, and/or publication of this article. All authors have read the submitted version of the manuscript.

**Disclosure:** The portion of the results presented in this manuscript have been presented at Congress of Neurological Surgeons congress in Houston, USA, in October 2024 (CNS ORAL PRESENTATIONS: 2024). Abstract was published as online: Alwakaa, Omar; Pojskic, Mirza MD; Bopp, Miriam; Nesim, Marco; Nimsky, Christopher MD, PhD; Saß, Benjamin. 272 Comparative Analysis of the Pedicle Screw Placement Accuracy, Revision Rate and Screw Loosening in a Single Center Using Fluoroscopy, iCT-Based Navigation and Robotic-Guided Surgery. Neurosurgery 71(Supplement_1): p 65, April 2025. | DOI: 10.1227/neu.0000000000003360_272 and is available online: September 2025).

**Acknowledgment:** We thank Emir Begagic, MD, for support in statistical analysis.
